# Electrical conductivity of melts: implications for conductivity anomalies in the Earth's mantle

**DOI:** 10.1093/nsr/nwab064

**Published:** 2021-04-12

**Authors:** Bao-Hua Zhang, Xuan Guo, Takashi Yoshino, Qun-Ke Xia

**Affiliations:** Key Laboratory of Geoscience Big Data and Deep Resource of Zhejiang Province, School of Earth Sciences, Zhejiang University, Hangzhou 310027, China; CAS Key Laboratory of Crust-Mantle Materials and Environments, School of Earth and Space Sciences, University of Science and Technology of China, Hefei 230026, China; Institute for Planetary Materials, Okayama University, Misasa 682-0193, Japan; Key Laboratory of Geoscience Big Data and Deep Resource of Zhejiang Province, School of Earth Sciences, Zhejiang University, Hangzhou 310027, China

**Keywords:** electrical conductivity, silicate melt, carbonate melt, high-conductivity anomaly, Earth's mantle

## Abstract

Magmatic liquids, including silicate and carbonate melts, are principal agents of mass and heat transfer in the Earth and terrestrial planets, and they play a crucial role in various geodynamic processes and in Earth's evolution. Electrical conductivity data of these melts elucidate the cause of electrical anomalies in Earth's interior and shed light on the melt structure. With the improvement in high-pressure experimental techniques and theoretical simulations, major progress has been made on this front in the past several decades. This review aims to summarize recent advances in experimental and theoretical studies on the electrical conductivity of silicate and carbonate melts of different compositions and volatile contents under high temperature and pressure. The electrical conductivity of silicate melts depends strongly on temperature, pressure, water content and the ratio of non-bridging oxygens to tetrahedral cations (NBO/T). By contrast, the electrical conductivity of carbonate melts exhibits a weak dependence on temperature and pressure due to their fully depolymerized structure. The electrical conductivity of carbonate melts is higher than that of silicate melts by at least two orders of magnitude. Water can increase electrical conductivity significantly and reduce the activation energy of silicate melts. Conversely, this effect is weak for carbonate melts. In addition, the replacement of alkali-earth elements (Ca^2+^ or Mg^2+^) with alkali elements causes a significant decrease in the electrical conductivity of carbonate melts. A distinct compensation trend is revealed for the electrical conductivity of silicate and carbonate melts under anhydrous and hydrous conditions. Several important applications of laboratory-based melt conductivity are introduced in order to understand the origin of high-conductivity anomalies in the Earth's mantle. Perspectives for future studies are also provided.

## Teaser

The electrical conductivity of melts depends strongly on temperature, pressure, volatile and structure, which is key to understand the origin of high-conductivity anomalies in the Earth's mantle.

## INTRODUCTION

Electrical conductivity is a powerful approach to deducing the temperature and chemical components of melts and fluids and their accumulation and distribution in the Earth's interior. Magnetotelluric surveys revealed the occasionally ubiquitous presence of high-conductivity anomalies in different tectonic environments, such as mid-ocean ridges [1], subduction zones [2,3] and volcanic regions [4,5]. Within Earth's mantle, electrical-anomaly zones overlap spatially with seismic ones at the lithosphere–asthenosphere boundary (LAB) [6,7], at the top of the 410 km discontinuity [8,9], and at the ultralow-velocity zone (ULVZ) of the core–mantle boundary (CMB) [10,11]. Partial melting, which is induced by volatile components (mainly H_2_O and CO_2_), has been used to explain these anomalies wholly or partly [2,6,7,12].

In Earth and terrestrial planets, magmatic liquids, including silicate and carbonate melts, are one of the principal carriers of mass and heat transfer. Knowledge of physical properties of magma at high pressures benefits the discussion and modeling of magmatism in various tectonic settings. Given that electrical conductivity is extremely sensitive to magmatic liquids, it can be an effective way to quantify melt and its distribution in order to interpret the low-velocity and high-conductivity anomalies observed in Earth's mantle.

The influences of temperature, pressure and composition on the electrical conductivity of silicate melts have been addressed by a number of laboratories [13–26]. However, investigations on hydrous [19,22,26–30] and carbonate melts [26,30–33] are still limited. Gaillard *et al.* [31] measured the electrical conductivity of carbonate melts, and they reported that 0.035–0.35 vol% of carbonate melts was sufficient to explain high-conductivity anomalies in the oceanic asthenosphere [1].

Given the high mobility of melts and their active reaction with surrounding substances during experiments, determining the conductivity of magmatic liquids at high pressure is difficult. Therefore, the chemical composition and volume fraction of melts in Earth's interior remains unclear. Efforts were exerted to advance experimental techniques of electrical conductivity measurement in magmatic liquids [34,35]. Meanwhile, melt structures and transport properties under high pressure were also investigated by theoretical predictions based on molecular dynamics (MD) simulations [36–45]. Experimental and theoretical studies together demonstrated significant changes in the structure and electrical properties of melts when the melt system contained water and carbon dioxide.

The combination of experimental measurements of electrical conductivity and magnetotelluric surveys has proven to be an excellent method for exploring the composition, status and dynamic processes within Earth's interior [1,2,12,26,30]. These methods deepen our comprehension of the behavior of magmas in Earth's interior greatly. This paper sketches out our current knowledge of electrical conductivity of silicate and carbonate melts obtained from experimental measurements and MD simulations and their dependence on temperature, pressure, compositions and volatile components. Empirical relationships among electrical conductivity parameters and important applications of experimental results are discussed. Finally, perspectives for future studies are suggested.

## Conductivity mechanisms in melts

Most of rock constitute materials act similarly to insulators at room temperature due to large energy gaps. However, they behave as semiconductors when the melting temperature is reached. Similarly to solid mantle minerals, the bulk conductivity of melts is attributed to different conduction mechanisms acting in parallel. These mechanisms are produced by the accumulative effects of moving charge carriers or defects with different valences and concentrations:

(1) \begin{equation*}\sigma {\rm{ = }}\sum\limits_i {{\sigma _i}} {\rm{ = }}\sum\limits_i {{N_i}} {z_i}{\mu _i},\end{equation*}

where *N_i_*, *z_i_* and *μ_i_* are the concentration, valence and mobility of the *i*th charge carrier, respectively. Usually, one or two ionic species with the highest mobility, such as Na^+^, dominate the conductivity in silicate melts [19,34,46]. The diffusive transport property of charge carriers contributes to ionic conductivity through the Nernst–Einstein equation:

(2) \begin{equation*}{\sigma _i}{\rm{ = }}\frac{{{D_i}z_i^2{N_i}}}{{{k_B}T{H_R}}},\end{equation*}

where *T* is the absolute temperature, *k_B_* is Boltzmann's constant, *D_i_* is the diffusion coefficient of the *i*th charge carriers and *H_R_* is the Haven ratio depending on the transport mechanism (usually 0.1 <* H_R_* < 1). The electrical conductivity and Na diffusivity closely follow the trend with a unified Haven ratio, supporting that an interstitial mechanism of Na transport should dominate silicate melts [47]. On the contrary, a small *H_R_* (<0.5) was observed in high-alkali glasses (i.e. Li_2_O-SiO_2_ and Na_2_O-SiO_2_), which implies that conductivity is significantly higher than tracer diffusivity [28].

## LABORATORY CONDUCTIVITY MEASUREMENTS OF MELTS

**Figure 1.**
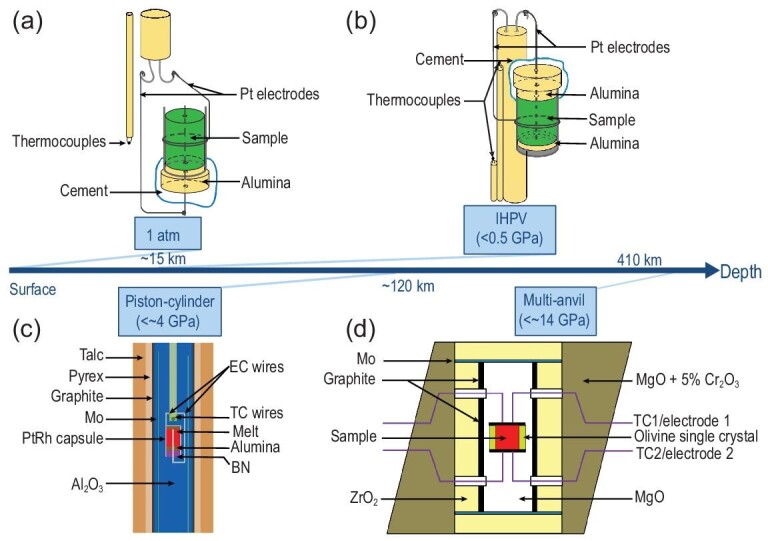
Schematic drawing of cell design for liquid conductivity measurement under different pressure ranges investigated. (a) A cylindrical capsule method at atmospheric pressure [19,22]. (b) Internally heated pressure vessel (IHPV) [22]. (c) Piston cylinder apparatus [27]. (d) Kawai-type multi-anvil apparatus [35]. More details about each cell can be found in the cited references. The pressure and corresponding depths achieved by each cell assembly are indicated by squares and the blue arrow, respectively. (a) and (b) were modified with permission from Wiley-VCH. (c) was modified with permission from Springer-Verlag GmbH.

**Figure 2.**
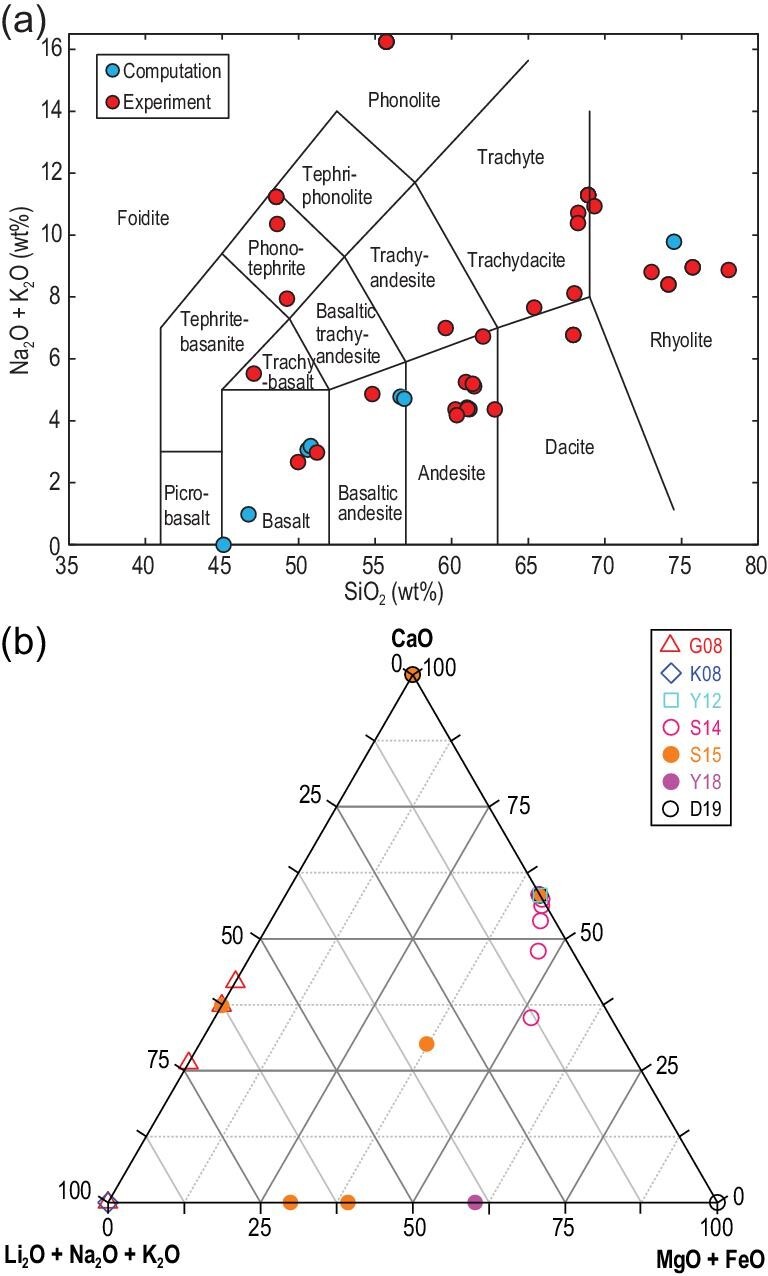
(a) The total alkali-silica (TAS) and (b) CaO–MgO+FeO–Li_2_O+Na_2_O+K_2_O ternary diagrams indicating chemical compositions of silicate melts [15,16,18,19,21–24,27,28,36,37,39–44,47–56] and carbonate melts [26,30–33,38,45,76], respectively, for which electrical conductivity data reported by experiment and MD simulation were used in this study.

Melts have a higher chemical activity than solids and can react easily with surrounding substances (i.e. electrodes and sample containers). Thus, electrical conductivity measurement of magmatic liquids is challenging. In addition, when water is incorporated into magmatic liquids, the sample dimensions of low-viscosity hydrous melts can easily change during measurement. Furthermore, maintaining the volatile content above the liquidus before and after conductivity experiments is difficult. To solve these difficulties and further understand the electrical properties of melts, experimentalists have made great efforts to establish reliable experimental methods in the past several decades. Presnall *et al.* [13] performed a preliminary study on the electrical conductivity of basaltic melts at 1 atm pressure using a hemispherical crucible method. On the basis of this technology, later works [19,22] adopted the coaxial cell design composed of a Pt tube as an external electrode and a Pt wire as an internal electrode (Fig. 1a). The cell assembly in Fig. 1a is a completely open system at 1 atm pressure. Thus, this experimental design cannot be used to measure the electrical conductivity of volatile-bearing (H_2_O and CO_2_) melts because volatiles readily escape in air. The electrical conductivity of hydrous melts has to be measured under high pressure in a closed environment to overcome this problem. Consequently, internally heated pressure vessel (<500 MPa) [22] (Fig. 1b), piston cylinder apparatus (<4 GPa) [27] (Fig. 1c) and Kawai-type multi-anvil apparatus (>5 GPa) [35] (Fig. 1d) were developed to measure the conductivity of magmatic liquids under high pressures based on the cell design established at 1 atm pressure. Using these unique experimental techniques, the electrical conductivity of silicate and carbonate melts with various compositions has been investigated up to 2173 K and 11 GPa [19,22,25–28,30,33,47,48]. Figure 2 and the Supplementary Data provide a compilation of chemical compositions for the reported conductivity data of basaltic [15,21–23,27,36,37,39,41,47,49], andesitic [15,22,23,36,37,42,47,50–52], dacitic [16,18,28,47,48,53], rhyolitic [16,19,36,37,54–56] and carbonate melts [26,30,31,33,38,45].

## OVERVIEW OF ELECTRICAL CONDUCTIVITY MEASUREMENT FOR SILICATE MELTS

### Temperature effect

**Figure 3.**
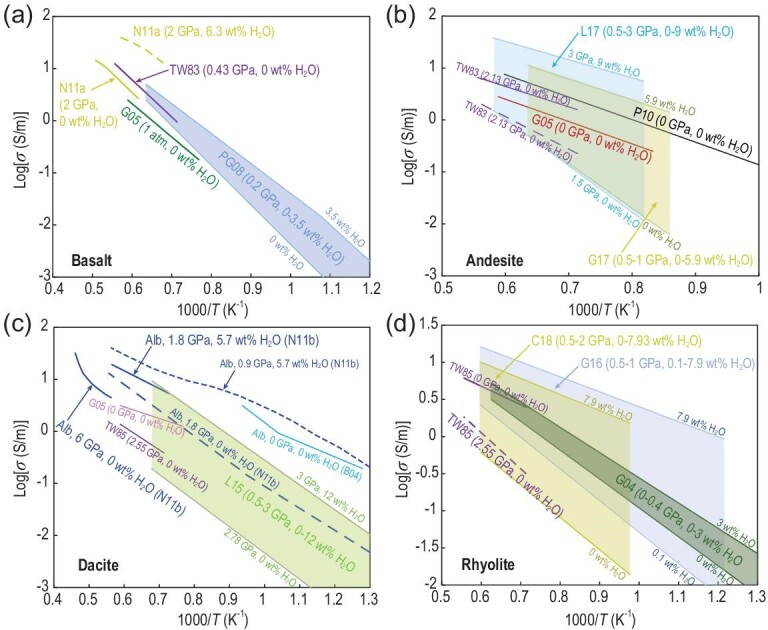
Electrical conductivity data for silicate melts. (a) Basalt. (b) Andesite. (c) Dacite. (d) Rhyolite. Data source: Basalt: TW83 [15], PG08 [22], N11a [27], G05 [47]; Andesite: TW83 [15], P10 [23], G05 [47], G17 [50], L17 [51]; Dacite: TW85 [16], B04 [18], G05 [47], N11b [28], L15 [48]; Rhyolite: TW85 [16], G04 [19], G16 [54], C18 [56].

Electrical conduction in liquid phases is a thermally activated process. Therefore, the electrical conductivity (*σ*) of silicate melts is primarily controlled by the concentration and mobility of ions. The markedly different conductive ions of silicate melts are a remarkable feature that is widely investigated (Fig. 2a). Melt conductivity increases with the increase in temperature. Given the complexity of electrical conduction in liquid phases, previous experimental investigations demonstrated two widely accepted formulae used in the electrical conductivity of silicate melts. The first one is a linear trend often found within certain temperature ranges in the plot of logarithmic conductivity versus inverse temperature (Fig. 3), and it is expressed by the Arrhenius relation:

(3) \begin{equation*}\sigma = {\sigma _0}\exp\! \left( { - \frac{{\Delta H}}{{RT}}} \right),\end{equation*}

where *R* is the gas constant, Δ*H* is the activation enthalpy and *σ*_0_ is the pre-exponential factor. In most cases, the dependence of electrical conductivity on temperature yields a single Arrhenius relation (Equation 3) (Fig. 3), which implies that Δ*H* shows no change with the increase in temperature. In contrast to the Arrhenius behavior, the temperature dependence of the electrical conductivity of hydrous basaltic [27] and albitic melts [18,28] shows a non-Arrhenius behavior (Fig. 3a and c, respectively). These types of non-Arrhenius data are frequently fitted by the empirical Vogel–Fulcher–Tammann (VFT) equation [57]: *σ = A*_VFT_exp[–*B*_VFT_/(*T*–*T*_0_)], where *A*, *B* and *T*_0_ are empirical constants. This behavior follows predictions from the relaxation of melt structure (i.e. the rearrangement of melt structure) [46,58]. The conductivity of hydrous basaltic melts (2 GPa, 6.3 wt% H_2_O) shows a downward curve above the glass transition temperature (*T*_g_), and Δ*H* gradually decreases for the liquid region compared with that for glass [27] (Fig. 3a). However, the conductivities of anhydrous albitic melts [18,28] exhibit a distinctly upward curve in the Arrhenius plot (Fig. 3c) with a large Δ*H* for the liquid region. These non-Arrhenian observations manifested that electrical conductivity and Δ*H* are not only strongly dependent on temperature but also melt composition, melt structure and water content. Moreover, such non-Arrhenian behavior may reflect changes in the conduction mechanism with temperature. Thermal motion becomes strong at high temperature given that charge carriers in melts collide with an increased number of other atoms. This finding was used to explain the non-Arrhenian behavior [27].

### Pressure effect

**Figure 4.**
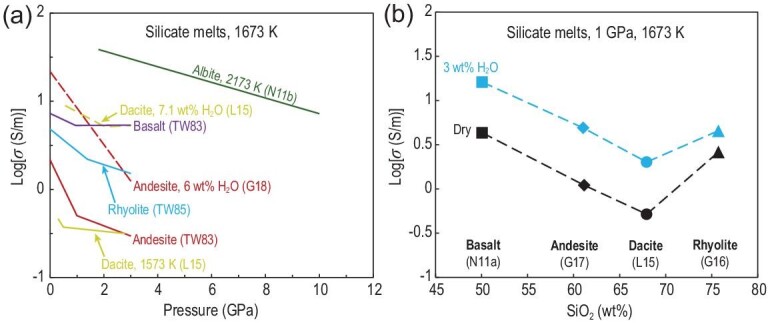
Influence of (a) pressure and (b) SiO_2_ on the electrical conductivity of silicate melts at 1673 K. Note that the black and blue symbols in (b) represent dry and hydrous (3 wt% H_2_O) melts, respectively. Data source: TW83 [15], TW85 [16], N11a [27], N11b [28], L15 [48], G17 [50], G16 [54], G18 [55].

An activation volume term (Δ*V*) is included in the Arrhenius relation to quantify the effect of pressure on electrical conductivity:

(4) \begin{equation*}\sigma {\rm{\ = \ }}{\sigma _0}\exp\! \left( { - \frac{{\Delta E{\rm{ + }}P\Delta V}}{{RT}}} \right),\end{equation*}

where Δ*E* is the activation energy. Δ*V* indicates the volume of moving species and is the most important parameter to determine the pressure effect of conductivity. In contrast to temperature, pressure generally imposes a negative effect on the electrical conductivity of melts (Fig. 4a). In the melt structure, atoms and ions are considered hard spheres. High ionic porosity at low pressure favors ionic transport, but a high pressure suppresses the proportion of ‘free volume’ (the volume without ions and atoms). Electrical conductivity has negative pressure dependence, which yields positive Δ*V.* In addition, electrical conductivity decreases with pressure, ranging from 3 cm^3^/mol to 30 cm^3^/mol [15,16,28,48,55]. Basaltic melt [15] has the smallest pressure effect, whereas rhyolitic [16], dacitic [48] and albitic melts [28] exhibit similar pressure dependence (Fig. 4a). Roughly, anhydrous andesitic melt [15] shows a large pressure effect at low pressure (<1 GPa), but this effect decreases at high pressure (>1 GPa). These observations reflect the effect of pressure on the degree of melt polymerization. Highly polymerized melts exhibit a strong pressure dependence [15,16,28,48,55].

### Melt composition effect

Network-forming cations (mainly Si and Al) and network-modifying metal cations (Na, K, Ca, Mg and Fe) construct polymerized oxide liquids with various degrees of polymerization in silicate melts [59]. Given that network-modifying cations are more mobile than network-forming ones [46], they are the most important charge carriers in anhydrous silicate melts. The electrical conductivity of silicate melts has strong dependence on chemical composition. Particularly, SiO_2_ content plays an important role in the electrical conductivity of silicate melts. As shown in Fig. 4b, the conductivity of dry and hydrous (3 wt% H_2_O) silicate melts gradually decreases with the increase in SiO_2_ content in the order of *σ*_Basalt_ > *σ*_Andesite_ > *σ*_Dacite_ at 1 GPa and 1673 K. However, the unusually high conductivity of rhyolitic melts should be closely related to the structure of silicate melts. The structure and composition of anhydrous silicate melts can be characterized by the ratio of NBO/T [59]. Melts with large NBO/T have a low degree of polymerization (or high degree of depolymerization) and Si content and a high conductivity. Silicate melts are generally ionic conductors [46], and their conductivity is dominated by several kinds of fast-moving ions, although all moving ions may contribute to electrical transport. In anhydrous melts, Na^+^ is suggested as the main charge carrier [19,27,47,48]. The contribution of  K^+^ (and other divalent cations) is limited because their diffusion coefficient is at least one order of magnitude lower than that of Na^+^ [60].

### Volatile effect

**Figure 5.**
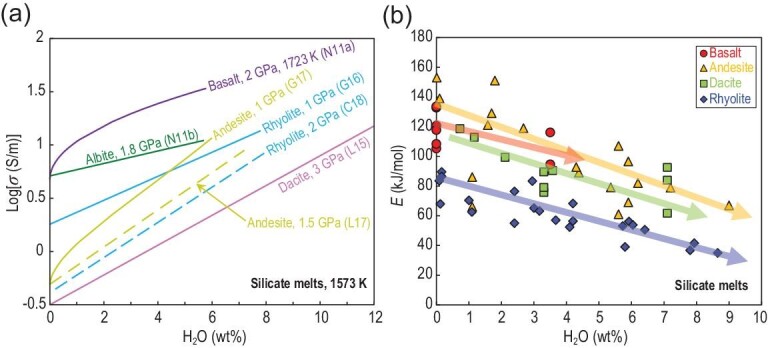
(a) Effect of H_2_O content on electrical conductivity of silicate melts at 1573 K. Data source: N11a [27], L15 [48], L17 [51], G16 [54], C18 [56]. (b) Variation of activation energy (*E* in kJ/mol) for silicate melts as a function of H_2_O content. All data are given in Fig. 2a and the Supplementary Data.

Water can significantly increase the electrical conductivity of silicate melts (Fig. 5a) [19,22,27,28,48,50,51,54,55]. The conductivity of dacite with 12 wt% H_2_O is ∼1.7 orders of magnitude higher than that of the dry one at 3 GPa [48]. Although pressure can decrease the electrical conductivity of silicate melts (Fig. 4a), its effect is less than that of water in the investigated experimental range (0.0 < H_2_O < 12 wt%; 0.15 < *P* < 3.0 GPa). The plot of log*σ* versus H_2_O content showed a linear relationship for rhyolitic [54–56], dacitic [48] and albitic melts [28] but a nearly parabolic one for andesitic [50] and basaltic melts [27] (Fig. 5a). This notable curvature in the logσ versus H_2_O plot implies that the influence of H_2_O is strong in the relatively low-H_2_O content range (<2 wt%), whereas the H_2_O effect on melt conductivity weakens with the increase in temperature and H_2_O content [27,48,50]. Such differences most likely arise from the role of H_2_O in enhancing liquid dynamics and the mobility of the majority of ionic species, rather than from direct contributions from protons or other hydrous species [19,46,48,50]. The 3 wt% H_2_O raises the electrical conductivity of Na-rich melts, such as albite and rhyolite, by less than a factor of two [28,54,56]; however, for basaltic and andesite melts with relatively low Na contents, this factor increases to approximately five [27,50,51] (Fig. 5a). Therefore, the strong H_2_O effect on conductivity can be attributed to the more effective mobilization of other charge carriers (e.g. Mg and Ca) by H_2_O in basaltic and andesitic melts than by Na [27,50].

On the other hand, as shown in Fig. 5b, from basaltic to rhyolitic melts, the Δ*E* of electrical conductivity decreases greatly with the increase in water content. Guo *et al.* [54] observed that the Δ*E* in rhyolitic melts at 1 GPa decreased from ∼81 kJ/mol for the anhydrous melt to ∼37 kJ/mol for the hydrous melt with 7.9 wt% H_2_O. By contrast, Gaillard [19] showed that increasing the H_2_O content can increase the conductivity but cannot change the activation enthalpy significantly in highly polymerized alkali-bearing silicate liquids. He concluded that the diffusion of Na remains as the conduction mechanism in water-rich liquids (3 wt% H_2_O) despite its relatively low atomic abundance. These observations suggest that the continuous decrease in the activation energy with the increase in H_2_O content can arise from continuous changes in the melt structure.

H_2_O-induced depolymerization of melt structure through the reaction H_2_O + O = 2 OH^–^, with O being a bridging oxygen (BO) ion [46,59], can enhance the movement of ions associated with depressed melt viscosity. This condition results in the decrease in activation enthalpy and overall increase in conductivity with added H_2_O [61]. Previous studies [19,62] indicated that hydrogen can enhance the mobility of charged carriers such as Na^+^, whereas the mobility of proton (H^+^) is notably lower than that of Na^+^ in alkali-bearing silicate melts [63]. Furthermore, OH^–^ diffuses slower than H_2_O by at least one order of magnitude [46,54]; in this situation, OH^–^ may contribute largely to the electrical conductivity of hydrous basaltic melt [64]. These findings suggest that increased electrical conductivity in hydrous silicate melts is mainly due to enhanced transport of Na, rather than structural relaxation [46,58], because the dissolution in water can decrease polymerization and increase the ionic porosity of melts [62].

**Figure 6.**
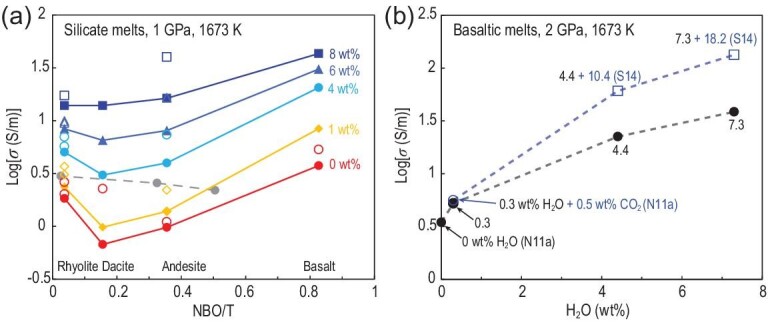
(a) Variation of electrical conductivity at 1673 K and 1 GPa (2 GPa for basalt) with silicate melt composition as characterized by the number of non-bridging oxygen ions per tetrahedrally coordinated cation (NBO/T) at different H_2_O contents. The colored symbols represent experimental data for basalt [27], andesite [51], dacite [48] and rhyolite [55]. The gray circles are the results of G05 [47]. The open symbols stand for the results reported by others [15,16,50,54,56]. (b) Influence of H_2_O and CO_2_ on the electrical conductivity of basaltic melt at 2 GPa and 1673 K. The black circles stand for hydrous basalt [27]. The blue circle represents basalt with 0.3 wt% H_2_O + 0.5 wt% CO_2_ [27]. The blue squares indicate carbonated hydrous basalt at 3 GPa [26]. The black and blue numbers are H_2_O and CO_2_ contents, respectively.

The primary influence of H_2_O on polymerization of silicate melts has been extensively studied. Water increases the ratios of free oxygen and NBO/T by decreasing the amount of BO [65,66]. Electrical conductivities of various silicate melts along the calc-alkaline series at 1673 K and 1 GPa were compared to demonstrate the influence of chemical composition (Fig. 6a). At ambient pressure, a decreasing trend from rhyolite through dacite to andesite was observed in dry silicate melts (closed gray circles in Fig. 6a) [47]. Although electrical conductivity increases with the increase in water content for each silicate melt, the enhanced magnitude varies with composition. From rhyolitic to basaltic melt, the conductivity decreases first and then increases when the H_2_O content is lower than 6 wt%. Dacitic melt exhibits the lowest electrical conductivity. With 8 wt% H_2_O, the conductivity increases monotonically by nearly half an order of magnitude from rhyolite to basalt melts with the decrease in polymerization (Fig. 6a). This finding agrees well with the considerable contribution from divalent cations and decreased viscosity of these melts in sequence [46,50]. Evidently, H_2_O has a more substantially pronounced effect on andesitic and basaltic melts than on silicic melts (Fig. 6a). Low viscosity and a high degree of depolymerization result in great ion mobility, which leads to the high electrical conductivity of basaltic melts [46]. In hydrous basaltic and andesitic melts, not only that of Na^+^ but also the mobility of divalent cations, such as Ca^2+^ and Mg^2+^, is remarkably enhanced. Consequently, these divalent cations provide significant contributions to the melt electrical conductivity, resulting in a pronounced increase in electrical conductivity for andesitic and basaltic melts compared to silicic ones [50,52]. The enhancement of the electrical conductivity of silicate melt by water is consistent with the findings indicating that its ionic porosity increases with the incorporation of H_2_O [67]. Therefore, the electrical conductivity of silicate melts is dominated by the mobility of conductive ions and ionic porosity of the melt.

In addition to H_2_O, the electrical conductivity of silicate melts may be influenced by CO_2_. Ni and Keppler [68] showed that silicate melts have a notably lower solubility in CO_2_ compared with H_2_O. Figure 6b shows a comparison of the electrical conductivity of basaltic melts with the same H_2_O content but different CO_2_ concentrations. When the CO_2_ concentration was below 0.5 wt%, Ni *et al.* [27] demonstrated that CO_2_ barely affected the electrical conductivity of basaltic melt (Fig. 6b). By contrast, Sifré *et al.* [26] revealed that the electrical conductivity of hydrous CO_2_-rich basaltic melts significantly increased with the increase in CO_2_ content (Fig. 6b). For different melt compositions, previous works generally showed negligible to moderately negative CO_2_ effect on viscosity [40,69]. Therefore, the weak CO_2_ effect on electrical conductivity observed by Ni *et al.* [27] is generally consistent with the viscosity behavior. In addition, previous works [39,40] have shown that carbonate groups in the melt structure can be divided into bridging CO_3_^2–^ (bonded with two network formers such as Si), non-bridging CO_3_^2–^ (bonded with one network former) and free CO_3_^2–^ (not bonded with network-forming cations such as Si and Al), with the last one being the dominant species in alkali-rich depolymerized melts. Morizet *et al.* [69] further proposed that although the formation of free CO_3_^2–^ enhances melt polymerization by converting non-BO to BO, melt viscosity may still decrease due to the formation of a carbonate subnetwork. Cancellation of these effects can explain the weak CO_2_ effect on melt viscosity and electrical conductivity. The weak dependence of electrical conductivity on low CO_2_ concentration (several wt% or less) also implies that CO_3_^2–^ is not an effective charge carrier [27]. With the further addition of CO_2_ (at values significantly greater than 10 wt%), the silicate network will eventually be disrupted, and CO_3_^2–^, with its increased mobility and concentration, can contribute significantly to electrical conduction, leading to extremely high electrical conductivity of carbonatite/carbonate melts.

On the basis of a compilation of available laboratory electrical databases, Pommier and Le-Trong [70] established a SIGMELTS. A web portal for electrical conductivity calculations in geosciences (code available from server at http://www.calcul.isto.cnrs-orleans.fr/sigmelts/) model to calculate the electrical conductivity of silicate and carbonate melts as a function of temperature, pressure, composition, water content and oxygen fugacity. Notably, the SIGMELTS model predicted higher electrical conductivity of anhydrous silicate melts more frequently than experimental measurements but obtained lower values for hydrous ones [50,55]. This difference between the SIGMELTS model and laboratory measurements could be due to the extremely limited experimental data on melts available at the time, which covered a narrow range of compositions and water contents. Another possible reason is that only Na^+^ was considered the dominant charge carrier in SIGMELTS [70]. Notably, other divalent cations (i.e. Mg^2+^ and Ca^2+^) may also contribute significantly to bulk conductivity under hydrous conditions [26–28,30,50,52,55], resulting in the underestimation of the conductivity of hydrous melts by SIGMELTS.

## OVERVIEW OF ELECTRICAL CONDUCTIVITY MEASUREMENT FOR CARBONATE MELTS

### Temperature and pressure effects

Carbonate melt is a key medium in the global deep carbon cycle and important for understanding related geochemical and geophysical processes, such as metasomatism [71], and low-velocity and high-conductivity anomalies observed in the asthenosphere [2,72,73] or other Earth interiors [74,75]. Although the occurrence and stability of carbonate-rich melts has been extensively studied, minimal constraints are placed on their physical properties, especially electrical conductivity at high temperature and pressure.

**Figure 7.**
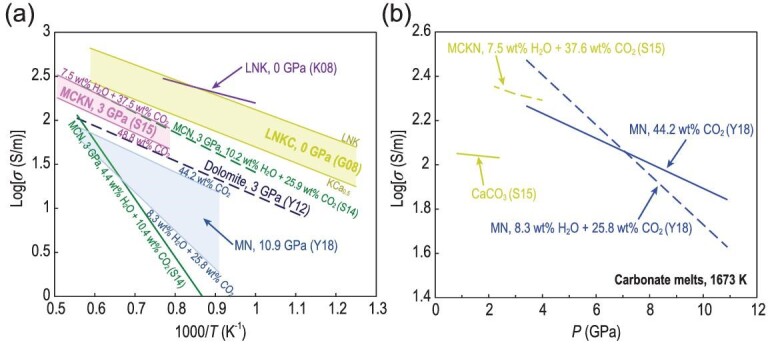
Electrical conductivity data for carbonate melts. (a) Temperature dependence. (b) Pressure dependence at 1673 K. Data source: S14 [26], S15 [30], G08 [31], K08 [32], Y18 [33], Y12 [76]. Note: LNK (Li_2_CO_3_-Na_2_CO_3_-K_2_CO_3_), LNKC (Li_2_CO_3_-Na_2_CO_3_-K_2_CO_3_-CaCO_3_), MN (MgCO_3_-Na_2_CO_3_), MCN (MgCO_3_-CaCO_3_-Na_2_CO_3_), MCKN (MgCO_3_-CaCO_3_-K_2_CO_3_-Na_2_CO_3_).

To date, several experimental studies [26,30–33] have investigated the electrical conductivity of carbonated melts, including single-alkaline carbonates (Li_2_CO_3_, Na_2_CO_3_ and K_2_CO_3_), natural calcite (CaCO_3_), dolomite (CaMg(CO_3_)_2_), magnesite (MgCO_3_) and their binary and ternary mixtures (Li_2_CO_3_-Na_2_CO_3_-K_2_CO_3_-CaCO_3_-MgCO_3_). Figure 7a shows the temperature dependence of electrical conductivity for molten single-phase carbonates and their mixtures. All experiments displayed the same behavior except for carbonate melts with low H_2_O and CO_2_ contents [26,33]. Their electrical conductivities exhibited a slight temperature dependence given that the values increased linearly with temperature by a factor of three within the investigated range (liquid region), suggesting a small activation enthalpy for the electrical conductivity of carbonate melts (∼20–70 kJ/mol). At atmospheric pressure, the electrical conductivity of Li-Na-K ternary carbonates, measured by Kojima *et al.* [32], was consistent with those reported by Gaillard *et al.* [31]. For anhydrous carbonate melts under high pressure, the conductivity of dolomite reported by Yoshino *et al.* [76] is comparable to that of MCKN (MgCO_3_-CaCO_3_-K_2_CO_3_-Na_2_CO_3_) [30] and MN (MgCO_3_-Na_2_CO_3_) [33] systems at 3 GPa (Fig. 7a). The difference in conductivity among these studies is within 0.1 log unit. These experimental observations [31,32] imply that the self-diffusion of alkali-elements (Li, Na and K) controls the electrical conductivity of alkali-carbonate melts, which is similarly observed in silicate melts [19,27,47].

For hydrous carbonate melts, Sifré *et al.* [26,30] observed that the electrical conductivity of carbonate melts increased with the increase in H_2_O and CO_2_ contents under asthenospheric pressure conditions (Fig. 7a). Their results indicated that the effect of H_2_O was strong at low water contents (<4 wt%); a weak effect was observed at high water contents. When the CO_2_ content in basaltic melt was lower than 8 wt%, the effect of CO_2_ was smaller than that of H_2_O, whereas it became significant when the CO_2_ content was above 8 wt%, exceeding the effect of water (Figs 6b and 7a). In contrast to the enhancement of conductivity in carbonate melts by H_2_O [26,30], Yoshino *et al.* [33] showed that H_2_O dramatically reduced the conductivity of carbonate melts and increased the activation enthalpy compared with that of the anhydrous MN (MgCO_3_-Na_2_CO_3_) (Fig. 7a), which may be caused by the low Na concentration in the carbonate samples.

Only Sifré *et al.* [30] and Yoshino *et al.* [33] performed electrical conductivity measurements at various pressures to evaluate the pressure effect on the conductivity of carbonated melts. Figure 7b shows that the electrical conductivity of anhydrous and hydrous carbonate melts decreased with the increased pressure at 1673 K. The conductivity of carbonate melts decreases with increased pressure, regardless of whether the carbonate melt has, or lacks, H_2_O. This negative pressure dependence yields positive Δ*V*. Notably, the Δ*V* (0.3 cm^3^/mol) determined by Sifré *et al.* [30] is smaller than that deduced from experiments [33] (1.8–3.6 cm^3^/mol). The smaller Δ*V* reported by Sifré *et al.* [30] was probably due to the limited pressure range (1–4 GPa) and experimental dataset, which prevented the precise determination of pressure effect on Δ*V*. Remarkably, Yoshino *et al.* [33] revealed that the pressure effect (Δ*V* = 3.6 cm^3^/mol) on hydrous carbonate melts is two times larger than that (Δ*V* = 3.6 cm^3^/mol) of anhydrous ones. In addition, they observed that the hydrous carbonate melt has a lower conductivity and higher Δ*H* compared with its anhydrous counterpart (Fig. 7a). As suggested by Yoshino *et al.* [33], a large decrease in the electrical conductivity of hydrous carbonate melts is possibly caused by the dehydration of samples during repeated heating at increasing pressure. In addition, the decrease in the content of highly mobile ions (particularly Na^+^) with pressure may result in a large negative pressure dependence because of the creation of electrically neutral species or the presence of slowly diffusing species when water is present. As a whole, pressure has a small effect on the electrical conductivity of carbonate melts due to their depolymerized structure and low ionic porosity.

### Melt composition effect

Carbonate melts are ionic liquids constituted by carbonate (CO_3_^2–^) molecular anions and metal cations. These ions interact mainly through coulombic interactions [59,77]. Given the electronic structure and intra-molecular bonding of carbonate ions, carbonate melts cannot polymerize to form network structures such as those observed with silicate melts [71]. As a result, the magnitude of the effect of chemical composition on the electrical conductivity of carbonate melts (Fig. 7) is considerably weaker than that on silicate melts (Fig. 3). Experimental observations [31,32] at ambient pressure demonstrated that small cation and low charge can lead to a high electrical conductivity. Thus, as alkali substitution follows the order Li > Na > K, increasing conductivity is observed for molten carbonates [62]. Furthermore, when alkali-earth elements (CaCO_3_ or MgCO_3_) replace alkali elements, they will trigger at least half an order of magnitude decrease in electrical conductivity [26,30,31,33] because of their lower self-diffusivities compared to alkali elements in silicate melts [62]. Dolomite [26,76] shows higher electrical conductivity and lower Δ*H* than calcite [26,30,38,45], indicating that Mg ions diffuse significantly faster than Ca ions. Therefore, bulk conductivity is dominated by the migration of alkali ions, which is similar in silicate melts. When CO_2_ was replaced by H_2_O, Sifré *et al.* [26] observed a weak increase in electrical conductivity, whereas Yoshino *et al.* [33] reported a slightly lower conductivity compared to that observed in anhydrous melts (Fig. 7a). Despite the opposite trend observed, water has a negligibly small effect on the electrical conductivity of carbonate liquids without a polymerized structure, given the small difference between anhydrous and hydrous melts.

## MD SIMULATIONS

### Silicate melts

When experiments faced difficulties with regard to the extreme conditions involved in the study of silicate melts, computer simulations became an attractive complementary approach. Advances in computer technology render classical (based on pairwise interatomic potential models) and first-principle MD (FPMD) simulations (based on density functional theory) feasible. MD and FPMD allow large and extended simulations of multi-component silicate melts [36,37,39–43,78–80]. Given the empirical or semi-empirical force fields used, their accuracy and extrapolation to high pressure–temperature range are often questioned. However, intensive computations provide hints on melt structures and dynamics with pressure, temperature and volatiles [44,66,81,82] and meaningful insights into how the bulk (macroscopic) properties of melts are controlled by atomic characteristics.

**Figure 8.**
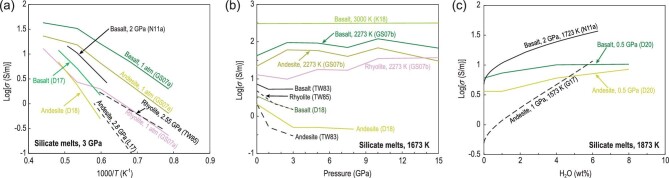
Comparison of molecular dynamics simulations with experimental data for electrical conductivity of silicate melts. (a) Log*σ* vs. 1/*T* at 3 GPa. (b) Log*σ* vs. *P* at 1673 K. (c) Log*σ* vs. H_2_O content at 1873 K. Data source: TW83 [15], TW85 [16], N11a [27], GS07a [36], GS07b [37], L17 [51], G17 [50], D17 [41], D18 [42], K18 [32], D20 [43]. Thin black lines indicate the experimental data.

Stein and Spera [83] carried out the first study of the conductivity of silicate melts using MD simulations. Their MD ionic conductivity data on NaAlSiO_4_-SiO_2_ melt under 4 ± 1.5 GPa and 2500–4500 K are consistent with the extrapolated experimental results at 490–730 K [84]. By implementing the MD simulation code with a simple interionic potential, Guillot and Sator [36] investigated the role of Na transport on the electrical conductivity of various silicate melts at 1 atm pressure. Their results showed that the highly depolymerized melts have high conductivities in the order of *σ*_Basalt_ > *σ*_Andesite_ > *σ*_Rhyolite_ (Fig. 8a). This trend has been observed in experimental studies on high-temperature liquids [15,16,27,47,51] as discussed above and consistent with recent MD research [40–44]. Vuilleumier *et al.* [39] performed FPMD simulations to quantify the influence of carbon dioxide (∼20 wt%) on the electrical conductivity of silicate melts at 2073 K and 12 GPa. Their FPMD simulations revealed that the charge distribution throughout the network was modified by the presence of carbonate ions (${\rm{CO}}_{\rm{3}}^{{\rm{2 - }}}$) in a certain manner, which greatly enhanced the electrical conductivity of carbonated basaltic melt [39]. Overall, as shown in Fig. 8a, MD simulations predicted that the temperature-sensitive variations of electrical conductivity for basalt [36,37,39,41], andesite [36,37,42] and rhyolite [36,37] melts at a given pressure are roughly consistent with experimental trends [15,16,23,27,47,50,51,55].

Figure 8b shows the comparison between the pressure dependence of the conductivity of silicate melts predicted from MD simulations, with those from experimental determinations. To our knowledge, most experiments on electrical conductivity of silicate melts were carried out below 3 GPa, except for those for albitic melts [28], which were conducted up to 10 GPa. At 1673 K, MD and experimental studies indicated that pressure has a negative effect on the electrical conductivity of silicate melts. Given the extremely limited pressure range, experimental determinations of Δ*V* showed a wide range of variations (0–24 cm^3^/mol) [15,16,19,22,28,48,50,54,55], which is considerably larger than those (<6 cm^3^/mol) deduced from MD simulations [37,40–42,44]. In addition, the calculated Δ*V* decreases with the increase in temperature [37,40–42,44]. Guillot and Sator [37], in calculating several silicate melts (e.g. basalt, andesite and rhyolite), have shown that the conductivity fluctuated slightly with the increase in pressure and finally increased slightly at 2273 K (Fig. 8b). At high temperature (3000 K), the basalt conductivity remains constant with the increase in pressure [44], indicating that Δ*V* is close to zero. Noticeably, MD studies [37,40–42,44] showed that pressure influences the conductivity of silicate melt at values

slightly below 15 GPa (Fig. 8a), which challenges the negative pressure dependence observed in experiments on rhyolitic to basaltic and ultramafic melts [15,16,28,48,55] (Fig. 4a).

One MD study focused on the H_2_O dependence of the electrical conductivity of silicate melts. Dufils *et al.* [43] performed comprehensive MD simulations to investigate the influence of H_2_O on the electrical conductivity of magmatic liquids by introducing a new interaction potential for H_2_O compatibility with a force field. As illustrated in Fig. 8c, at fixed pressure and temperature, H_2_O dependence on melt conductivity predicted from MD calculations [43] is notably weaker than those reported from experimental measurements [27,50]. This difference may be attributed to the different melt compositions and/or pressure ranges between experimental studies and MD simulations.

### Carbonate melts

**Figure 9.**
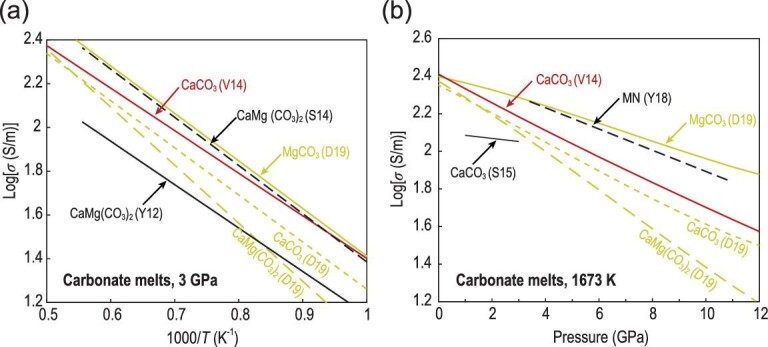
Comparison of molecular dynamics simulations with experimental data for electrical conductivity of carbonate melts. (a) Log*σ* vs. 1/*T* at 3 GPa. (b) Log*σ* vs. *P* at 1673 K. Data source: S14 [26], S15 [30], Y18 [33], V14 [38], D19 [45], Y12 [76]. Thin black lines indicate the experimental data.

Unlike silicate melts, MD simulations of the electrical conductivity of carbonate melts are scarce. To date, two studies [38,45] have reported the electrical conductivity of MgCO_3_, CaCO_3_ and CaMg(CO_3_)_2_. Figure 9a demonstrates that the electrical conductivity of CaCO_3_, which was predicted from an MD simulation by Vuilleumier *et al.* [38], was ∼0.15 log unit higher than that calculated by Desmaele *et al.* [45] at low temperatures; however, this small difference diminishes at high temperatures. In addition, the temperature dependence of the conductivity of dolomite (CaMg(CO_3_)_2_) reported by Yoshino *et al.* [76] is roughly consistent with that predicted by Desmaele *et al.* [45] at 3 GPa, but at least half an order of magnitude lower than that calculated by Sifré *et al*. [26]. Remarkably similar to experimental observations in carbonate melts [26,30–33] (Fig. 7a), a small temperature dependence yields a low activation enthalpy for the electrical conductivity of carbonate melts (∼30–50 kJ/mol) [38,45] (Fig. 9a).

Pressure decreases the electrical conductivity of carbonate melts (Fig. 9b). The Δ*V* of 0.3 cm^3^/mol for CaCO_3_ reported by Sifré *et al.* [30] is considerably smaller than those (∼2.5 [38] and 3.3 cm^3^/mol [45]) predicted from MD simulations. However, the value of Δ*V* (1.8 cm^3^/mol) for the anhydrous Mg–Na carbonate system determined by Yoshino *et al.* [33] is comparable to those (2.1–2.6 cm^3^/mol) of MgCO_3_ and CaMg(CO_3_)_2_ calculated by Desmaele *et al.* [45].

### Discrepancies between experiments and computations

On the basis of the above discussions, available data on the electrical conductivity of silicate and carbonate melts are largely inconsistent among different laboratories, various computations, and between laboratory measurements and MD simulations (Figs 3–9). Notably, the inconsistencies in experimental data reported by different researchers have led to large differences in the inferred melt fraction and composition of the mantle and created great confusion for the geoscience community. From the viewpoint of laboratory measurements, the possible causes of these existing discrepancies and uncertainties can be attributed to three aspects. (i) Experimental techniques (oxygen fugacity control and sample contamination). Early pioneering experiments [13,19,22] were performed in air under 1 atm pressure (Fig. 1a) or in an internally heated pressure vessel (<500 MPa) [22] (Fig. 1b). In such set-ups, the capsule is unsealed, and thus, argon is likely to incorporate the sample in a gas pressure vessel. As a result, redox conditions under low pressure are uncontrolled and determined. Therefore, the effect of oxygen fugacity on conductivity is unknown, which ultimately affects conductivity because oxygen fugacity will alter the speciations of H_2_O and CO_2_ in melts. In addition, metal capsules and electrodes (especially Pt) have been used to measure melt conductivity (Fig. 1a–c). In this situation, the composition of a Fe-bearing melt sample is expected to change due to the formation of Fe-Pt alloy induced by the reaction between the sample and capsule/electrode, which eventually leads to large uncertainties in experimental results. Other influencing factors include sample deformation at high temperature and pressure conditions. (ii) Very limited pressure range. As mentioned above, almost all conductivity experiments were conducted below 4 GPa, except for two studies [28,33]. As shown in Figs 4 and 7–9, a low experimental pressure revealed the large uncertainty about the influence of pressure (activation volume) on melt conductivity [15,16,19,22,28,48,50,54,55]. (iii) Volatile (H_2_O and CO_2_) content determination. Precise determination of H_2_O and CO_2_ contents in quenched glass before and after conductivity measurement is a key procedure to assess whether the melt has changed. However, this step is a challenging task. In most experiments, instead of being actually measured, CO_2_ content was assumed to remain constant as with the starting material during the conductivity measurement. Importantly, the concentrations and speciations of H_2_O and CO_2_ in melts may need to be cross-checked for accuracy and validity by multiple methods, such as Fourier transformation infrared spectroscopy, Raman spectroscopy and secondary-ion mass spectrometry. The solution for the above problems will be conducive to the establishment of a quantitative and reliable conductivity model for magmatic liquids as a function of temperature, pressure, composition and oxygen fugacity.

In the case of computation, most discrepancies possibly resulted from the small difference between potential models provided by different researchers. Thus, the accuracy and applicability of MD simulations over extended temperature–pressure space are often questionable because of the empirical or semi-empirical nature of the force field used. Moreover, current theoretical calculations are only feasible for certain ideal simple systems [36–43,45]. Limited studies have comprehensively considered the influence of multiple factors, including a wide range of temperatures, pressures, volatile components, chemical compositions and oxygen fugacities, on the conductivity of melt simultaneously. The elucidation of the speciation and incorporation mechanism of H_2_O and CO_2_ in magmatic liquids will be an important topic for future simulation studies, to reconcile the quantitative discrepancies between MD results and experimental data. We anticipate more first-principle computational studies and laboratory measurements under high pressure to improve our knowledge about the behavior and dynamics of silicate and carbonate melts in the temperature–pressure conditions of the whole mantle.

## COMPARISON OF ELECTRICAL CONDUCTIVITY OF SILICATE AND CARBONATE MELTS

**Figure 10.**
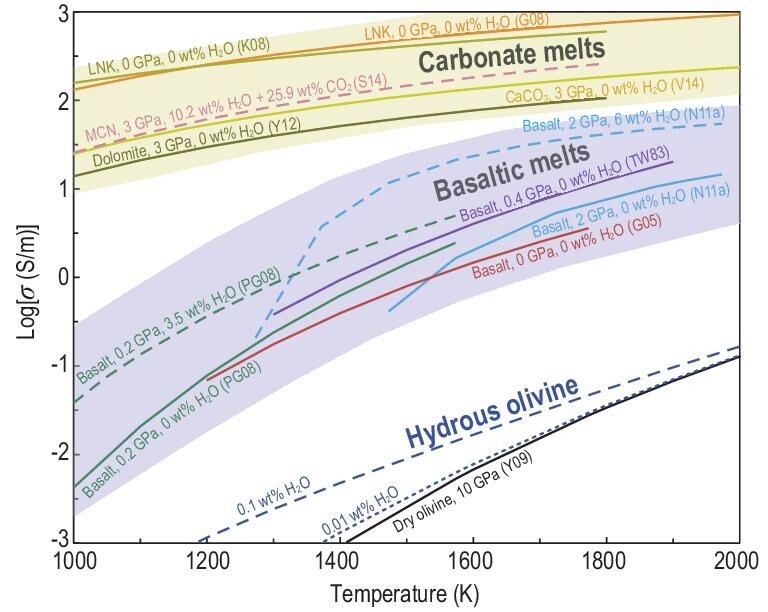
Comparison of electrical conductivity of carbonate melts, basaltic melts and hydrous olivine. The solid and dashed curves are for anhydrous and hydrous melts, respectively, with the labels indicating the weight percentage of water and pressure. Data source: PG08 [22], N11a [27], S14 [26], G08 [31], K08 [32], V14 [38], G05 [47], Y12 [76], Y09 [85]. The dark yellow and light purple areas indicate the range of the conductivity in carbonate melts and basaltic melts, respectively.

Figure 10 compares experimental studies of the electrical conductivity of basaltic melts, carbonate melts, and dry and hydrous olivine systems. Electrical conductivities of carbonate melts with different compositions are in the range of 10^1^–10^3^ S/m at different temperatures and pressures [26,30–33,38,45,76], and these values are up to two orders of magnitude higher than those of basaltic melts [15,22,23,27,47] and almost five orders of magnitude higher than those of pure mantle olivine [85]. From the viewpoint of melt structure, silicate and carbonate melts are fairly different. However, network-forming iono-covalent silicate liquids (e.g. silica) and ionic carbonate liquids (e.g. molten salts) constitute two endmembers of a continuum. Polymerization degree (i.e. NBO/T) is used to characterize the former. However, the latter is fully depolymerized, and its liquid structure is controlled by the size ratio between anions and cations and by their valence state. The electrical conductivity and viscosity of silicate and carbonate melts are thermally activated, diffusion-related processes. The diffusion of electric charge species with the highest mobility, such as alkali ions, governs electrical conductivity, whereas the diffusion of species-forming melt frameworks (Si or O for silicate minerals) governs rate-control viscosity. Given the ultralow viscosity of depolymerized carbonate melts [86], the diffusion coefficient between each ionic species is expected to have a smaller difference than that for alkali silicate melts. Based on precise experimental studies and MD simulations, the most striking discovery is the markedly different roles of water in the conductivity of silicate and carbonate melts. In silicate melts, water facilitates the movement of Na^+^, which is the main charge carrier, and leads to an elevated electrical conductivity [19,22,27,28,43,48,50,51,55] and a decrease in Δ*H* (Fig. 5b). By contrast, water has a negligible effect on carbonate melts because of the lack of a polymerized structure that inhibits the diffusion of all cations [26,30]. Instead, the introduction of water slightly reduces the conductivity and increases the Δ*H* of carbonate melts [33].

## COMPENSATION LAW

**Figure 11.**
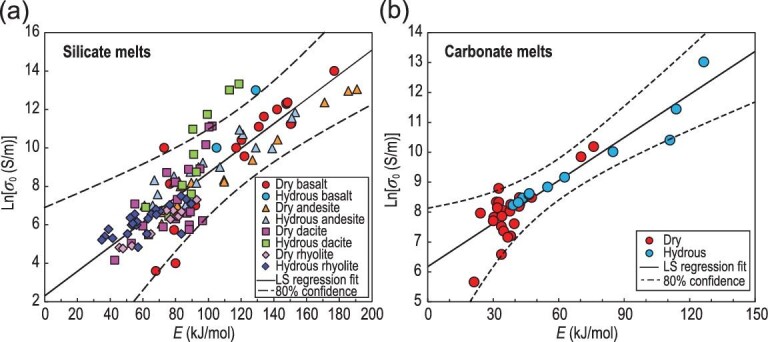
Compensation plots of activation energy *E* (in kJ/mol) versus the natural logarithm of pre-exponential factor ln*σ*_0_ (*σ*_0_ in S/m) for electrical conductivity in (a) silicate melts and (b) carbonate melts. The data and relevant references are given in Fig. 2 and the Supplementary Data.

Tyburczy and Waff [15] observed a positive linear relationship between ln*σ*_0_ (in S/m) and *E* (in kJ/mol) for the electrical conductivity of molten andesite and basalt; this relationship is usually called the ‘compensation effect’ [87]. This effect has also been observed in solid phases [88,89]. Remarkably, a detailed overall review of all published experimental and theoretical data (Fig. 2) reveals a distinct compensation trend for the electrical conductivity in silicate (Fig. 11a) and carbonate melts (Fig. 11b) under anhydrous and hydrous conditions. The compensation relations are as follows:

(5) \begin{eqnarray*} &&{\rm{Silicate}}\,\,{\rm{melts}}\!:\,\,\ln {\sigma _0} = 2.302(0.324)\nonumber\\ &&\qquad +\,\, 0.064(0.003) \times E,\end{eqnarray*}

(6) \begin{eqnarray*} &&{\rm{Carbonate}}\,\,{\rm{melts}}\!:\,\,\ln {\sigma _0} = 6.175(0.207)\nonumber\\ &&\qquad+\,\, 0.048(0.004) \times E,\end{eqnarray*}

where *E* is the activation energy in kJ/mol, and *σ*_0_ is the pre-exponential factor in S/m. Given the compensation effect, each conducting species or conduction mechanism converges to a constant conductivity (*σ*_C_) at the characteristic temperature *T*_C_. The calculated *T*_C_ and *σ*_C_ were 1879 ± 84 K and 10 ± 4 S/m for silicate melts, respectively. The values were 2507 ± 193 K (*T*_C_) and 481 ± 111 S/m (*σ*_C_) for carbonate melts. In this observation, the activation energies *E* covered a notably wide range (∼20–200 kJ/mol) and pre-exponential factors ln*σ*_0_(∼3–14 S/m). Therefore, the compensation effect for silicate and carbonate melts deduced in this study cannot result from experimental artifacts. In general, all charge carriers (or defects) contribute to the total electrical conductivity when they are present. However, under given thermodynamic conditions, only one or two types of defects dominate. This condition has been proven by the experimentally measured conductivity of silicate melts [22,35]. Figure 11 presents all electrical conductivity data for silicate and carbonate melts that satisfy the same compensation law. They were obtained at different melt structures, chemical compositions, water contents and pressures. This observation provides further evidence that the compensation law for electrical conductivity is due to the same conduction mechanism (with mobile cations as the main charge carriers) in each melt.

## ORIGIN OF HIGH-CONDUCTIVITY ANOMALIES IN EARTH’S MANTLE

Magnetotelluric investigations revealed an anomalous high-electrical-conductivity region beneath the oceanic asthenosphere near the East Pacific Rise [1,90,91] (order ∼10^–1.0^ S/m), below the north Pacific at a depth of 200–250 km [92] in the subduction zones [2] (order ∼10^–0.5^ S/m) and in the lithospheric mantle [75,93] (order ∼10^–1.0^–10^–1.2^ S/m). Although the origin of these high-conductivity anomalies remains debated, the presence of melt is the most likely cause for such magnetotelluric observations. This conjecture has been reinforced by numerous electrical conductivity studies based on laboratory experiments [25–27,30,31,33,48,51,76,94–96]. In addition, high-conductivity anomalies are closely associated with low seismic velocities in numerous locations [3,97]. The presence of an interconnected network of a conductive phase within a granular matrix, such as a fluid or melt, dominates the bulk conductivity of a rock and masks the conductivity of the matrix phase. Similarly, the presence of wetting liquids can significantly reduce seismic wave velocities [98,99]. Nevertheless, the interpretation of the magnitude of geophysical signals from low-velocity and high-conductivity anomalies largely depends on melt fraction and composition.

In the case of partially molten samples, not only the conductivity of melts and solid phase but also the detailed morphology of partial melt must be determined. Various geometry models based on simplified melt distributions, such as the cube model [100], tube model [101] and Hashin–Shtrikman upper (HS+) bound model [102], were proposed to evaluate the influences of these factors on bulk electrical conductivity. These models consider the different distribution geometries of melt in samples, either along the grain boundaries and triple junctions or in isolated pockets.

Waff [100] proposed a cube model to describe bulk conductivity as a function of the conductive-phase fraction. This model supposes that cubic grains with low conductivity are all of the same size and surrounded by a high-conductivity phase (*σ_m_*) layer of uniform thickness. The conductivity is dependent on the conductive-phase fraction (*φ*), and the conductivity of the resistive phase (*σ_solid_*) is negligibly small. The effective conductivity (*σ_bulk_*) according to this model is given by the following:

(7) \begin{equation*}{\sigma _{bulk}}{\rm{\ = \ }}\left[ {1{\rm{\ - \ (1 \ - \ }}\phi {{\rm{)}}^{{\rm{2/3}}}}} \right]{\sigma _m}.\end{equation*}

The tube model proposed by Schmeling [101] represents the case that conductive phase is not distributed along the grain boundaries but in a network along the triple junctions. The bulk conductivity is described by the following:

(8) \begin{equation*}{\sigma _{bulk}}{\rm{ \ = \ }}\frac{{\rm{1}}}{{\rm{3}}}\phi {\sigma _m} + {\rm{(1 \ - \ }}\phi {\rm{)}}{\sigma _{solid}}.\end{equation*}

The HS+ bound [102] is a frequently used model in predicting the maximum bulk conductivity of a matrix consisting of a conductive phase surrounding spherical inclusions with low conductivity. In this model, spherical grains are isolated from each other by the conductive phase. Thus, this model is applicable to cases in which the conductive phase distributes along the grain boundaries and fills the triple junctions of spherical grains. In the HS+ model, the bulk conductivity is expressed as follows:

(9) \begin{eqnarray*}\sigma _{bulk}^{\rm HS + }{\rm{\ = \ }}{\sigma _m} + \frac{{1 - \phi }}{{{{({\sigma _{solid}} - {\sigma _m})}^{ - 1}} + (\phi /3{\sigma _m})}}.\nonumber\\ \end{eqnarray*}

The cube model, tube model and HS+ bound model provide estimates for a well-interconnected melt phase. The tube model gives a high melt fraction for a given electrical conductivity value and hence represents the upper limit of the melt fraction determined from electrical conductivity [35,103]. However, the effective conductivity deduced from the cube model is notably close to that calculated from the HS+ bound.

**Figure 12.**
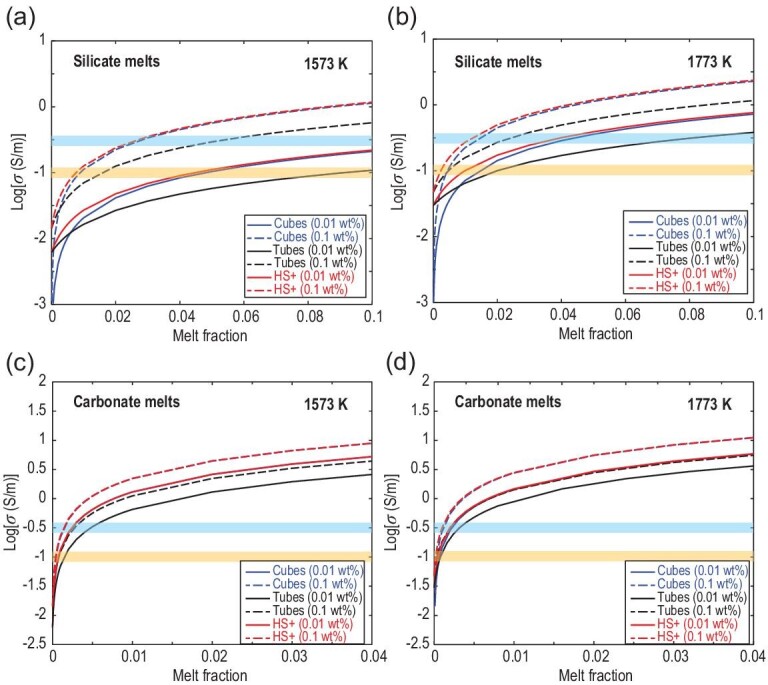
Predicted bulk conductivity of the partial molten peridotites as a function of the melt fraction at 3 GPa, based on the cube model [100], the tube model [101] and Hashin–Shtrikman upper bound model [102]. (a) Olivine + basalt system at 1573 K. (b) Olivine + basalt system at 1773 K. (c) Olivine + carbonate melt system at 1573 K. (d) Olivine + carbonate melt system at 1773 K. Calculations assume a partition coefficient of 0.002 for water between olivine and the melt. The cyan and orange shaded regions indicate high conductivity anomalies in the subduction zones [2] (∼10^–0.5^ S/m) and in the upper mantle [1,75,90–93] (∼10^–1.0^ S/m).

To better constrain the melt fraction in Earth's mantle, the bulk conductivities of partially molten samples with different water contents were calculated using the cube model, tube model and HS+ bound model at 1573 and 1773 K for olivine + silicate and olivine + carbonate melts, respectively. The water partitioning coefficient [104,105] was assumed to be $D_{{{\rm{H}}_{\rm{2}}}{\rm{O}}}^{{\rm{Olivine/melt}}}$ = 0.002, and the electrical conductivity of olivine was referenced from the work of Yoshino *et al.* [85]. The conductivity data of basaltic and carbonate melts were reported by Ni *et al.* [27] and Yoshino *et al.* [33], respectively. Figure 12 illustrates how the electrical conductivity of H_2_O-poor (with 0.01 wt% H_2_O) and H_2_O-rich (with 0.1 wt% H_2_O) mantles responds to the increased degree of melting at 3 GPa and 1573 and 1773 K, together with geophysically observed high-conductivity anomalies in the upper mantle. Given that the conductivity of silicate melt depends strongly on temperature, the bulk conductivities for the olivine + silicate melt system, which were inferred from the three models mentioned above, at 1573 K (Fig. 12a) are considerably lower than those at 1773 K (Fig. 12b). By contrast, the calculated electrical conductivities of olivine + carbonate melt from the three geometric models yielded similar values at 1573 and 1773 K (Fig. 12c and d, respectively), regardless of the water-poor or water-rich condition. As an example of silicate melt, the high-conductivity region with ∼10^–1.0^ S/m near the East Pacific Rise [1,90,91] can be explained by 0.6–1.0 vol% hydrous silicate melts inferred from the cube model and HS+ bound model at 1573 K (Fig. 12a); this range agrees with that estimated in the laboratory conductivity measurement under shear deformation [94,106] and seismic velocity experiments [98,99], and is consistent with that (0.25–1.25 wt%) proposed by Kawakatsu *et al.* [6] at the LAB. On the other hand, an abnormally high conductivity (10^–0.5^ S/m) with a small anisotropy (0.3 log units) beneath the Cocos Plate at the Middle America trench (45–70 km in depth) [2] may be interpreted as a small fraction of silicate melts containing more H_2_O and CO_2_ components and relatively low temperature as suggested by Zhang and Yoshino [95].

Carbonate melts with two orders of magnitude more electrical conductivity than silicate melts (Fig. 10) have been invoked to explain anomalous conductivity in deep regions of the mantle asthenosphere. Gaillard *et al.* [31] argued that electrically conductive mantle regions, which are thought to be caused by water-bearing olivine or silicate melts, can also be explained by the presence of low-volume (0.1%) carbonate melts. Sifré *et al.* [26] demonstrated that carbonatitic incipient melts of carbonate-bearing peridotite can reproduce high conductivities and low seismic velocities in the upper part of the asthenosphere. Remarkably, long-period magnetotelluric soundings revealed a high-conductivity region (10^–1.0^ S/m) in the continental lithospheric mantle beneath the Slave craton [93] at depths of 80–120 km, below the Brazilian craton [75] at depths of 100–150 km, and in the oceanic lithospheric mantle beneath the northwestern Pacific [92] at depths of 200–250 km. Experimental petrology studies demonstrated that carbonate melts can only be stable at depths below 75 km (>2.5 GPa) [107,108]. If a small dihedral angle of olivine–carbonate system is considered (20°–30°) [109,110], then a small amount of interconnected hydrous carbonated melts can reasonably explain the high-conductivity anomalies in the deep part of the continental and oceanic lithospheric mantle. In explaining the conductivity value of 10^–1.0^ S/m [75,92,93] (Fig. 12c and d), the estimated melt fraction of carbonate is ∼0.01–0.03 vol% at 1573 or 1773 K regardless of the water content, and this value is higher than that (0.005 vol%) proposed by Pinto *et al.* [75]. However, the melt fraction is one-third to one order of magnitude lower than that (0.1 vol%) predicted by Gaillard *et al.* [31], who first argued that a small amount of carbonate melts leads to elevated conductivity of the low-velocity zone beneath the oceanic lithosphere. This estimate is roughly consistent with those (0.03–0.2 vol%) suggested by Sifré *et al.* [30] and Gardés *et al.* [111] but notably lower than that (0.1–0.5 vol%) proposed by Yoshino *et al.* [25,33,76]. However, estimation of the amount of melt in the Earth's interior remains a huge challenge, because electrical properties of melt are affected by various factors, such as temperature, pressure, water content, chemical composition and melt morphology. In particular, precise knowledge of the oxidation state in the deep mantle is needed in order to consider the viability of the carbonate melt hypothesis, because the speciation and mobility of volatile elements are affected by oxygen fugacity [14,24,44]. Under oxidized conditions, carbon is present as carbonate or carbonatite melt, which is potentially mobile and can lower the mantle solidus by several hundred degrees. However, under reduced conditions, carbon is present as graphite or diamond, which is immobile and cannot affect the melting temperature [107,108]. Studies of natural peridotite xenoliths showed that oxygen fugacity decreases toward deep Earth [112]; thus, carbonatite melts or carbonatites cannot be at depths deeper than ∼120 km in the subcratonic and asthenospheric mantle [72,113]. In addition, extremely small amounts of graphite or diamond (no carbonate melt) are expected to be present in garnet lherzolites beneath cratons with extremely low oxygen fugacity [74]. Nevertheless, the graphite film on grain boundaries cannot be maintained stably over long geological periods due to the high interfacial energy between silicate minerals [114,115] and is thus not a likely candidate to account for high-conductivity anomalies in Earth's mantle.

The foregoing discussions showed that to explain the high-conductivity anomalies in the Earth's mantle, one would need 0.6–1.0 vol% silicate melts or 0.01–0.03 vol% carbonate melts. However, the amount of melt in the mantle and its composition remain an open question. Experimental petrologies [116,117] and theoretical studies [118,119] demonstrated that substantial partial melting is limited to the vicinity of mid-ocean ridges, and the amount of melt produced in the asthenosphere away from the ridge is small (∼0.1% or less). Notably, the melt fraction of ∼0.1% or less is too small to explain low seismic wave velocities although it is close to the value necessary to explain electrical conductivity, which is also considerably lower than that (∼0.5% to several percent) estimated in laboratory measurements [25–27,30,31,33,48,51,76,94–99,106] and geophysical observations [1,3,6,75,90–93]. When partial melt is invoked to explain high-conductivity anomalies, another important factor to consider is whether the temperature in Earth's interior is high enough to produce and maintain melting. A recent study on the thermal conductivity of granitoids [120] suggested that partial melting due to dehydration of hydrous minerals can occur in the shallow depths of the thickened crust of the Tibetan Plateau. Thus, a comprehensive understanding of melt fraction and composition in Earth's mantle, or the thorough interpretation of magnetotelluric or seismological profiles, requires systematic multidisciplinary constraints that are not limited to geophysical, geochemical, petrological and high-pressure experimental methods. In addition to the partial melt model, the importance of solid-state mechanisms to enhance electrical conductivity and reduce seismic wave velocities has been noted. Faul and Jackson [121] demonstrated that the low-velocity zone in the upper mantle can be explained by olivine without the presence of partial melting or any fluids. Similarly, Karato and Wang [122] suggested that the high-conductivity anomalies revealed by magnetotelluric surveys in Earth's mantle can be well explained by solid-state mechanisms, i.e. hydrous olivine and its high-pressure polymorphs. This idea is also supported by the electrical conductivity of granulites [123], pyroxenites [124] and eclogites [125,126].

## CONCLUDING REMARKS AND FUTURE PERSPECTIVES

This paper provides a critical review of the available conductivity data of silicate and carbonate melts from laboratory measurements and MD simulations. The following is a summary of our present understanding on several issues concerning the electrical conductivity of magmatic liquids.

- Silicate melt has a polymerized structure, and the degree of polymerization is characterized by NBO/T. The order of polymerization degrees is rhyolite < dacite < andesite < basalt. The smallest NBO/T implies the highest degree of polymerization, along with the high amount of Si and large ionic porosity, implying a great pressure dependence. By contrast, carbonate melts have a fully depolymerized structure, resulting in low ionic porosity and small pressure effect.
- The electrical conductivity of silicate melts greatly increases with the increase in temperature, whereas that of carbonate melts is weakly dependent on temperature.
- Pressure can modify the melt structure and decrease the mobility of ionic species, thus indicating a negative effect on the electrical conductivity of silicate and carbonate melts.
- Water can significantly increase the electrical conductivity and reduce the Δ*H* of silicate melts; conversely, its influence on the electrical conductivity of carbonate melts is weak and promotes the increase in their Δ*H*. The conductivity of silicate and carbonate melts is exclusively controlled by alkali ions.
- The electrical conductivities of carbonate melts are at least two orders of magnitude higher than those of silicate melts. Given that alkali substitution follows the order Li > Na > K, molten carbonates present increasing conductivity. In addition, the replacement of alkalis by alkali-earth elements (Ca^2+^ or Mg^2+^) causes a significant decrease in the electrical conductivity of carbonate melts.

Although considerable progress has been made on the electrical conductivity of melts by high-pressure experiments and MD calculations, controversies and confusions are abundant among different research groups. The discrepancies of data may arise from the differences in experimental arrangements or techniques, such as the standard material, measurement circuit and measurement frequency, and the potential empirical models adopted in MD calculations. In most cases, great caution must be implemented in the utilization of laboratory conductivity data and various geometric models for actual application in Earth's interior. In particular, the following aspects need to be strengthened further.

- With the great advancement in laboratory measurement techniques, the electrical conductivity of magmatic liquids has been initially measured by the two-electrode method using a single-frequency alternating-current signal and then by the four-electrode method employing a sweeping-frequency impedance analyzer since the 1990s. Nevertheless, the reliability of laboratory measurements and the accuracy of experimental results still need to be further improved, especially at high pressure. Moreover, finding a suitable standard substance for the background insulation resistance test is crucial in establishing a baseline or benchmark for laboratory conductivity measurements under high-temperature and high-pressure conditions, and it can be used to reconcile the discrepancies among different laboratories.
- Although the SIGMELTS model provides a simple and fast calculation approach on the electrical conductivity of silicate and carbonate melts as mentioned above, this model lacks precision due to the limitations of the SIGMELTS itself, which is based on limited experimental data and covers a narrow range of compositions and water contents. Therefore, a universally applicable conductivity model must be established for single-phase and/or two-phase systems, or multiphase systems as a function of temperature, pressure, composition, water content and oxygen fugacity.
- A comprehensive and accurate understanding of melt composition, volume fraction, distribution and dynamics in Earth's interior and a thorough interpretation of magnetotelluric or seismological observations requires a systematic multidisciplinary approach, including not only laboratory measurements of the transport properties (electrical conductivity, seismic velocity) of melts under high temperature and pressure and geochemical analyses, but also geophysical observations and theoretical simulations.
- Pressure has a great effect on the structure and electrical conductivity of magmatic liquids. It causes changes in the melt structure and thus induces significant pressure dependence. However, the current maximum pressure for the electrical properties of silicate melts and carbonate melts is up to 10 [28] and 10.9 GPa [33], respectively. The electrical conductivity of magmatic liquids must be measured over a wide pressure range of up to at least 24 GPa to better understand the low-velocity and high-conductivity anomalies in deep Earth, such as atop the 410 and 660 km discontinuity, and even ULVZ and CMB.
- The effects of volatile components (H_2_O and CO_2_) and oxygen fugacity on the transport properties of magmatic liquids at high temperature and pressure conditions are still not well understood, especially for carbonate melts. Notably, the electrical conductivity of silicate and carbonate melts with extremely high water content (up to 12 wt% H_2_O [48]) has not been reported thus far. When the water content is sufficiently high, supercritical fluids may be formed in Earth's interior [127,128], especially in the subduction zone. Thus, laboratory measurement of the electrical conductivity of supercritical fluids needs to be studied in the future.
- Computer simulation (MD or FPMD) is an effective approach to elucidate the speciation and incorporation mechanism of volatiles (H_2_O and CO_2_) in silicate and carbonate melts. However, most existing theoretical studies were performed on melt systems with relatively simple compositions, especially for carbonate melts [38,45]. More simulation studies on the melt composition and thermodynamic conditions of the real mantle are required to reconcile the quantitative discrepancies between MD results and experimental data.

Improvement on the above issues will provide new insights into the thermodynamics, transport and other physical and chemical properties of magmatic liquids in deep Earth.

## Supplementary Material

nwab064_Supplemental_File
